# Predictors of acarbose therapeutic efficacy in newly diagnosed type 2 diabetes mellitus patients in China

**DOI:** 10.1186/s40360-022-00621-2

**Published:** 2022-10-18

**Authors:** Rong Zhang, Quanxi Zhao, Rong Li

**Affiliations:** 1 Department of Geriatrics, Xijing Hospital of Air Force Medical University, No.127 West Changle Road, Xi’an, 710032 China; 2 Department of Pharmacy, Second People’s Hospital of Shaanxi Province, No.3 Shangqin Road, Xi’an, 710068 China

**Keywords:** Acarbose, Newly diagnosed type 2 diabetes mellitus, Therapeutic efficacy, Predictors

## Abstract

**Background:**

Acarbose is one of the optimal drugs for patients with the first diagnosis of type 2 diabetes mellitus (T2DM). But what kind of emerging patients has the best therapeutic response to acarbose therapy has never been reported. To this end, we investigated predictors of acarbose therapeutic efficacy in newly diagnosed T2DM patients in China.

**Methods:**

A total of 346 T2DM patients received acarbose monotherapy for 48 weeks as part of participating in the Study of Acarbose in Newly Diagnosed Patients with T2DM in China (MARCH study) from November 2008 to June 2011. Change in glycated hemoglobin (ΔHbA1c) served as a dependent variable while different baseline variables including sex, age, disease duration, weight, body mass index (BMI), systolic blood pressure (SBP), diastolic blood pressure (DBP), HbA1c, fasting plasma glucose (FPG), 2-h postprandial blood glucose (2 h PG), fasting insulin (FINS), 2-h postprandial insulin (2 h INS), early insulin secretion index (IGI), homeostasis model assessment of insulin resistance index (HOMA-IR), homeostasis model assessment of beta cell function (HOMA-B), area under the curve (AUC) of glucagon, insulin and GLP-1 were assessed as independent predictors. Step-wise multiple linear regression was employed for statistical analysis.

**Results:**

The results suggested that independent predictors of ΔHbA1c at 12 weeks included baseline body weight (*β* = − 0.012, *P* = 0.006), DBP (*β* = 0.010, *P* = 0.047), FPG (*β* = 0.111, *P* = 0.005) and 2 h PG (*β* = 0.042, *P* = 0.043). Independent predictors of ΔHbA1c at 24 weeks included disease duration (*β* = 0.040, *P* = 0.019) and FPG (*β* = 0.117, *P* = 0.001). Finally, independent predictor of ΔHbA1c at 48 weeks was disease duration (*β* = 0.038, *P* = 0.046).

**Conclusions:**

Acarbose may be more effective in newly diagnosed T2DM patients with low FPG, low 2 h PG and obesity. The earlier T2DM is diagnosed and continuously treated with acarbose, the better the response to therapy.

## Background

Acarbose is the first ɑ-glucosidase inhibitor used in the clinical therapy of type 2 diabetes mellitus (T2DM). It may delay the absorption of carbohydrates in the intestine to reduce postprandial blood glucose [1, 2]. Although acarbose has been launched in China for more than 20 years, it still dominates the hypoglycemic drugs. The main reasons are that acarbose shows significant efficacy for Chinese population which take Carbs as their main diet; In addition, acarbose has fewer side effects and is safe in the elderly, which is especially suitable for China, an aging country [3–5]. In China, the first head-to-head study comparing the therapeutic efficacy of acarbose and metformin (MARCH study) confirmed that acarbose was non-inferior to metformin in HbA1c reduction for the initial therapy of T2DM patients [6]. This study suggested acarbose was a suitable alternative for the initial therapy of newly diagnosed T2DM. However, the influence of baseline characteristics of T2DM patients on the response to acarbose is seldomly considered. The identification of screening factors affecting the prognosis to acarbose could be helpful in clinical practice. Although the MARCH study confirmed the non-inferiority of acarbose to metformin in terms of HbA1c reduction, which subgroup of the T2DM population has the best therapeutic response to acarbose therapy has never been reported. This study explored the prognostic factors affecting the therapeutic efficacy of 48-week acarbose monotherapy in newly diagnosed T2DM patients in China.

## Methods

### Patients

A total of 393 T2DM patients received acarbose monotherapy for 48 weeks as part of the MARCH study. However, 47 of these patients were excluded from this study due to drug side effects, lack of efficacy, combination therapy with insulin secretagogues and other factors. The most common side effects were mild to moderate gastrointestinal symptoms. Only six patients occurred serious adverse events. But there were no deaths or severe hypoglycaemic events [6]. The remaining 346 patients were included for statistical analysis in this study. The study included 210 men and 136 women, ranging in age from 30 to 70. The inclusion and exclusion criteria of the MARCH study were previously published [6]. Informed consent was obtained before participation in the study. The study protocol was approved by the Ethics Committees of 11 hospitals and registered with Chinese Clinical Trial Registry (No. ChiCTR-TRC-08000231) [6].

### Procedures

MARCH study was a 48-week, randomised, open-label, non-inferiority trial. After a 4-week lifestyle modification run-in, patients were assigned to 24 weeks of monotherapy with sustained-released metformin hydrochloride up to 1500 mg, once daily, or up to 100 mg of acarbose, three times daily and followed by 24-week add-on therapy with insulin secretagogues if needed [6]. Primary endpoints were reduction in HbA1c at week 24 and week 48 timepoints. This study was a post hoc analysis of the MARCH study. All the variables were obtained from the database of MARCH study. Change in glycated hemoglobin (ΔHbA1c) at week 12, 24 or 48 timepoints served as the treatment outcome or a dependent variable while different baseline variables including sex, age, disease duration, weight, BMI, SBP, DBP, HbA1c, FPG, 2 h PG, FINS, 2 h INS, IGI, HOMA-IR, HOMA-B, AUC_glucagon_, AUC_insulin_, AUC_GLP-1_ were assessed as independent predictors.

### Statistical analysis

Normally distributed data were expressed as mean ± standard deviation, whereas data without a normal distribution as median (25–75% interquartile). Correlation analysis was performed with Pearson^’^s or Spearman rank correlation analysis. Multivariate analysis was performed with step-wise multiple linear regression. The independent variables were primarily screened with *P* < 0.15 as a criterion for univariate analysis. Independent variables with statistical significance were included in the multiple linear regression analysis. The variance inflation factor (VIF) was used to assess multicollinearity of independent variables. A VIF of > 10 suggests the presence of multicollinearity; variables with a VIF of > 10 was excluded from the regression model. SPSS version 22.0 was employed for statistical analysis and a value of *P* < 0.05 was considered statistically significant.

## Results

### Baseline characteristics of the patients

The characteristics of newly diagnosed T2DM patients who received acarbose monotherapy for 48 weeks were analysed. The average age of the patients was 50.4, the average disease duration was 1.5 months. Other descriptive variables are shown in Table 1.

**Table 1:** Baseline characteristics of 346 T2DM patients treated with acarbose

Variables	Values
Sex (male/female)	210/136
Age (years)	50.4 ± 9.2
Disease duration (months)	1.5 (1.1–2.8)
Weight (kg)	70.0 ± 10.6
BMI (kg/m^2^)	25.5 ± 2.7
SBP (mmHg)	124.1 ± 12.5
DBP (mmHg)	79.5 ± 8.8
HbA1c (%)	7.5 ± 1.2
FPG (mmol/L)	8.2 ± 1.5
2 h PG (mmol/L)	12.5 ± 2.8
FINS (mU/L)	12.6 ± 9.1
2 h INS (mU/L)	34.1 ± 18.6
IGI	2.6 (1.2–4.7)
HOMA-IR	3.7 (2.4–5.9)
HOMA-B	48.2 (28.8–75.7)
AUC_glucagon_ (pg/mL*min)	12,127 (9673–15,970)
AUC_insulin_ (uIU/mL*min)	4421 (3142–6240)
AUC_GLP- 1_ (pmol*min)	2743 (1725–4320)

*BMI* body mass index, *SBP* systolic blood pressure, *DBP* diastolic blood pressure, *HbA1c* Glycated hemoglobin, *FPG* fasting plasma glucose, *2 h PG* 2-hour postprandial blood glucose, *FINS* Fasting insulin, *2 h INS* 2-hour postprandial insulin, *IGI* Early insulin secretion index, *HOMA-IR* homeostasis model assessment of insulin resistance index, *HOMA-B* homeostasis model assessment of beta cell function, *GLP- 1* Glucagon-like peptide- 1, *AUC* Area under the curve. AUC (insulin, glucagon, and GLP- 1) was calculated by trapezoid method from the four-point curve as 0 min, 30 min, 120 min, and 180 min. AUC was calculated with the following equations (for example): AUCinsulin = (INS0min + INS 30 min) × 30/2 + (INS30 min + INS120 min) × 90/2 + (INS120 min + INS180 min) × 60/2. HOMA index was calculated with the following formulae: HOMA-IR = FINS×FPG/22.5; HOMA-B = 20 × FINS/(FPG-3.5); IGI = (INS30-FINS)/(PG30-FPG) [6]

### Correlation of ΔHbA1c with baseline variables

Correlation analysis showed that the ΔHbA1c at 24 and 48 weeks was negatively correlated with baseline HbA1c, FPG and 2 h PG, but positively correlated with baseline early insulin secretion index (IGI) (*P* < 0.05; Table 2).

**Table 2:** Correlation of ΔHbA1c with baseline variables

Variables	ΔHbA1c at week 24		ΔHbA1c at week 48	
	r	P	r	P
Age (years)	0.066	0.242	−0.021	0.715
Disease duration (months)	0.056	0.321	0.095	0.102
Weight (kg)	−0.035	0.536	−0.017	0.773
BMI (kg/m^2^)	−0.067	0.238	−0.075	0.201
SBP (mmHg)	0.011	0.845	−0.017	0.766
DBP (mmHg)	0.073	0.195	0.063	0.279
HbA1c (%)	−0.784	< 0.001	− 0.762	< 0.001
FPG (mmol/L)	−0.270	< 0.001	− 0.309	< 0.001
2 h PG (mmol/L)	−0.318	< 0.001	− 0.339	< 0.001
FINS (mU/L)	−0.040	0.482	0.054	0.368
2 h INS (mU/L)	0.043	0.450	0.002	0.976
HOMA-IR	− 0.074	0.201	−0.069	0.249
HOMA-B	0.097	0.091	0.115	0.054
IGI	0.180	0.002	0.150	0.012
AUC_glucagon_ (pg/mL*min)	−0.093	0.104	−0.095	0.107
AUC_insulin_ (uIU/mL*min)	0.099	0.089	0.023	0.698
AUC_GLP- 1_ (pmol*min)	−0.013	0.823	−0.049	0.406

*ΔHbA1c* reduction in HbA1c

### Multivariate analysis of factors affecting ΔHbA1c

With ΔHbA1c serving as the dependent variable, a number of potential predictor variables were examined, including sex, age, disease duration, BMI, SBP, DBP, HbA1c, FPG, 2 h PG, FINS, 2 h INS, HOMA-IR, HOMA-B, IGI, AUC_glucagon_, AUC_insulin_ and AUC_GLP-1_. The results showed that, after adjustment of other variables, baseline FPG, 2 h PG, body weight, and DBP were independent predictors of ΔHbA1c at 12 weeks (*P* < 0.05; Table 3). FPG and disease duration were independent predictors of ΔHbA1c at 24 weeks (*P* < 0.05 for all). Finally, disease duration was independent predictor of ΔHbA1c at 48 weeks (*P* < 0.05).

**Table 3:** Stepwise multiple regression analysis of ΔHbA1c predictors

Variables	Partial regression coefficient	Standard error	Standardized regression coefficient	t	P
**Model 1**					
FPG (mmol/L)	0.111	0.039	0.146	2.805	0.005
Weight (kg)	−0.012	0.004	−0.112	−2.758	0.006
2 h PG (mmol/L)	0.042	0.021	0.109	2.036	0.043
DBP (mmHg)	0.010	0.005	0.080	1.995	0.047
**Model 2**					
FPG (mmol/L)	0.117	0.034	0.146	3.416	0.001
Disease duration (months)	0.040	0.017	0.087	2.351	0.019
**Model 3**					
Disease duration (months)	0.038	0.019	0.081	2.005	0.046

Model 1: With 12-week ΔHbA1c as the dependent variable, the *R*^2^ of the regression equation was 0.586 and the constant was 3.609; Model 2: With 24-week ΔHbA1c as the dependent variable, the *R*^2^ of the regression equation was 0.589 and the constant was 4.001; Model 3: With 48-week ΔHbA1c as the dependent variable, the *R*^2^ of the regression equation was 0.546 and the constant was 4.653

## Discussion

Acarbose is presently one of the most common anti-diabetic drugs in clinical practice. Its anti-diabetic effect is dependent on the intake of dietary carbohydrates. Thus, the therapeutic efficacy of acarbose is superior in Chinese T2DM patients who consume a carbohydrate-dominant diet. In the present study, the predictors of therapeutic response following 48 weeks of acarbose therapy among patients with newly diagnosed T2DM who participated in the MARCH study were evaluated. The investigation of patient factors (including blood glucose level, BMI etc.) affecting therapeutic efficacy of drugs can lead to the identification of predictors of therapeutic efficacy, thereby leading to more individualized and more efficacious therapy. In the present study, the correlation between various baseline variables and therapeutic efficacy of acarbose was evaluated in 346 patients with newly diagnosed T2DM.

Our results suggest that more factors affect the short-term efficacy, which was consistent with the anticipated results. Low FPG and low 2 h PG suggest a mild disease or an early stage of T2DM. Under this condition, the impairment of islet β cell function is mild, and thus patients have a better response to acarbose. Obesity was another predictor of ΔHbA1c at 12 weeks. Some studies have shown that acarbose has advantages in reducing body weight of T2DM patients [7, 8]. Indeed, the MARCH study also revealed that acarbose had a better capability in reducing body weight by comparison to metformin [6]. The underlying reason for weight loss could be that acarbose decreases appetite and opposes unwanted fat storage by reducing food intake. Our study confirmed that acarbose was more suitable for obese patients with newly diagnosed T2DM.

Stepwise multiple regression analysis further illustrated that the number of factors predicting the clinical efficacy of acarbose was reduced over time. Short disease duration was a consistent predictor of ΔHbA1c. Thus, early intervention is critical in helping to correct disordered glucose metabolism in T2DM patients. In addition, patients achieving greater reductions in HbA1c had a lower IGI. Correlation analysis showed that ΔHbA1c was positively related to IGI. There is evidence showing that IGI is transiently inhibited in the hyperglycemic state [9, 10], and lifestyle intervention or use of anti-diabetic drugs to control blood glucose is able to reverse IGI [11, 12]. In early phase of T2DM, low IGI suggests that it is possible to reverse impaired β cell function. Thus, low IGI at baseline is clinically important for the prediction of clinical efficacy of acarbose monotherapy.

Only patients with newly diagnosed T2DM were recruited into the present study and may not represent the general population of T2DM patients. The sample size of this study is relatively small, and there may be bias. Thus, further studies with larger sample sizes are warranted to confirm our findings. In addition, the follow up in the present study was performed for only 48 weeks. In future studies, long term follow up is required to evaluate and predict the long-term efficacy of acarbose monotherapy.

## Conclusion

Factors that can be used to predict the short-term efficacy of acarbose monotherapy among patients with newly diagnosed T2DM include baseline body weight, DBP, FPG and 2 h PG. In addition, patients with low FPG, low 2 h PG, and obesity may achieve a better efficacy of acarbose monotherapy. And the earlier T2DM is diagnosed and continuously treated with acarbose, the better the response to therapy.

## Data Availability

The data that support the findings of this study are available from MARCH study. The raw data was obtained with permission of Prof. Wenying Yang, the leader researcher of MARCH study [6].

## Abbreviations

T2DM: type 2 diabetes mellitus
MARCH: Acarbose in Newly Diagnosed Patients with Type 2 Diabetes Mellitus in China
ΔHbA1c: change in glycated hemoglobin
DBP: diastolic blood pressure
FPG: fasting plasma glucose
PG: postprandial blood glucose
VIF: variance inflation factor
IGI: early insulin secretion index
BMI: body mass index
SBP: systolic blood pressure
FINS: fasting insulin
2 h INS: 2-hour postprandial insulin
HOMA-IR: homeostasis model assessment of insulin resistance index
HOMA-B: homeostasis model assessment of beta cell function
AUC: area under the curve
